# Low-risk individuals with primary biliary cholangitis and significant liver stiffness: prognosis and treatment

**DOI:** 10.1007/s12072-024-10743-w

**Published:** 2024-12-11

**Authors:** Dawei Ding, Yinan Hu, Gui Jia, Boling Wang, Linhua Zheng, Juan Deng, Ruiqing Sun, Xiufang Wang, Guanya Guo, Lina Cui, Yulong Shang, Ying Han

**Affiliations:** State Key Laboratory of Holistic Integrative Management of Gastrointestinal Cancers, National Clinical Research Center for Digestive Diseases, Xijing Hospital of Digestive Diseases, The Air Force Military Medical University, Xi’an, 710032 Shaanxi China

**Keywords:** The GLOBE score, Liver stiffness, Prognosis, Risk stratification

## Abstract

**Background:**

Some patients treated with ursodeoxycholic acid (UDCA) or combined fenofibrate had well-controlled biochemical parameters but high liver stiffness, and the prognosis as well as therapeutic options for these patients may be an area worthy of further exploration.

**Aims:**

To explore the prognosis and treatment of patients with low-risk and high liver stiffness.

**Methods:**

A retrospective study included 424 cases of UDCA monotherapy and 102 cases of combined fenofibrate treatment.

**Results:**

The combination of liver stiffness measurement (LSM) and the GLOBE score improved prognostic prediction for patients with UDCA monotherapy (area under the receiver operating characteristic curve [AUC] of 0.868 (0.811–0.925) for the fitted model and 0.834 (0.767–0.900) for the GLOBE score, *p* = 0.006). Further analyses revealed that LSM had an additive prognostic effect mainly in low-risk patients defined by GLOBE < 0.5 (AUC, 0.777 [0.724–0.825] vs 0.642 [0.583–0.699], *p* = 0.001). For patients in the low-risk group, the prognosis was worse when LSM > 11 kPa (7/53 [13%] vs 2/227 [1%], *p* = 0.001). The prognosis was consistent between patients in the “low-risk and LSM > 11 kPa” group and the medium-risk group defined by 0.5 < GLOBE < 1.8 (7/53 [13%] vs 22/121 [18%], *p* = 0.418). In low-risk patients treated with combined fenofibrate therapy, the prognosis was worse when LSM > 11 kPa (3/21 [14%] vs 0/47 [0%], *p* = 0.022). The prognosis was consistent between patients in the “low-risk and LSM > 11 kPa” and the medium-risk groups (3/21 [14%] vs 6/27 [22%], *p* = 0.353). Antifibrotic drugs failed to reduce the incidence of the primary outcome (5/45 [11%] vs 5/27 [19%], *p* = 0.598), and delayed the progression of LSM in patients with low-risk and LSM > 11 kPa at 36 months of follow-up (changes in LSM, − 3.31 [− 5.04 to − 1.52] vs − 1.74 [− 2.83 to 1.5], *p* = 0.046).

**Conclusions:**

Patients with GLOBE-defined low-risk and LSM > 11 kPa had a poor prognosis, and antifibrotic therapy may slow the progression of liver stiffness in these patients.

**Supplementary Information:**

The online version contains supplementary material available at 10.1007/s12072-024-10743-w.

## Introduction

Primary biliary cholangitis (PBC), a gradually progressing autoimmune liver disease, inevitably progresses to cirrhosis and potentially fatal complications when inadequately managed [1]. Ursodeoxycholic acid (UDCA), the prevailing standard therapy for PBC [2, 3], ensures extended periods without liver transplantation (LT) or death, irrespective of the disease’s stage or the observed biochemical response [4]. However, about one-third of PBC patients do not fully respond to UDCA, primarily because their elevated alkaline phosphatase (ALP) or bilirubin levels persist after a year of treatment. This group of patients continues to face a high risk of unfavorable long-term health outcomes [5, 6].

The Rotterdam [7], Toronto [8], Paris-I [9], and Paris-II [5] criteria have been employed to distinguish between patients who respond to UDCA treatment and those who do not, after periods of 12, or 24 months. Despite their use, these criteria retain certain limitations due to the intricate nature of PBC [10]. The GLOBE score [11], which takes into account baseline measurements and parameters post-1 year of UDCA therapy, have been developed and implemented to forecast the long-term prognosis in patients with PBC.

In the present day, among the array of liver elastography methods, vibration-controlled transient elastography stands out as one of the most favored and extensively utilized techniques globally [12]. The use of VCTE for liver stiffness measurement (LSM) has been confirmed as an easy and dependable method for diagnosing liver cirrhosis or significant fibrosis in a variety of chronic liver conditions [13, 14]. Moreover, it has been correlated with an increased risk of portal hypertension, liver decompensation, hepatocellular carcinoma, and liver-related mortality across various liver diseases, including PBC [14–17].

In clinical practice, some patients treated with UDCA or combined fenofibrate have well-controlled biochemical markers but a high degree of liver stiffness, and the prognosis and therapeutic options for these patients are unclear. In the present retrospective study, we used the GLOBE score to define low-risk individuals and the LSM to stratified risk, and explored potential treatment strategies in low-risk patients with high LSM.

## Materials and methods

### Study population

We analyzed 424 cases of UDCA monotherapy and 102 cases of combined fenofibrate treatment at Xijing Hospital of Digestive Diseases (Xi’an, Shaanxi, China) from January 2016 to April 2024. PBC can be diagnosed by meeting any two of the following three criteria based on the guidelines of the European Association for the Study of the Liver [3]: (1) elevated ALP, an indicator of cholestasis, and imaging that rules out extrahepatic or intrahepatic bile duct obstruction; (2) positive anti-mitochondrial antibody (AMA)/AMA-M2 or positive for anti-sp100 and/or anti-gp210; (3) pathology with typical evidence of PBC.

The inclusion criteria were: (1) standardized UDCA and fenofibrate treatment; (2) receiving at least one LSM test, at which time UDCA or combination fenofibrate therapy administered for at least 6 months.

The exclusion criteria were (1) comorbid viral hepatitis, alcohol-related liver disease, drug-induced liver injury, autoimmune hepatitis, metabolic dysfunction associated fatty liver disease, or hereditary liver disease; (2) missing vital data; (3) treatment with glucocorticoids; and (4) comorbid malignancy (excluding tumors that have been cured), or prior LT.

UDCA and fenofibrate were administered orally at doses of 13–15 mg/kg/day and 200 mg/day, respectively. Patients were treated with additional fenofibrate based on whether they meet the Toronto criteria (alkaline phosphatase is less than 1.67 times the upper limit of normal) after 6 months of UDCA treatment. For patients with poor response, fenofibrate therapy was added.

### Study design

A retrospective cohort study was conducted. First, we confirmed that LSM has an additional prognostic role in the GLOBE score. Second, we used the GLOBE score to categorize the included cases into high, medium, and low-risk groups (0.5 and 1.8) [15], and to assess which part of the included cases LSM specifically had an additional prognostic effect on. Third, we explored the cut-off value of LSM to optimize the risk stratification of low-risk group. Fourth, we evaluated the effect of this cut-off value on patients treated with combined fenofibrate therapy. Finally, we explored the effect of antifibrotic drugs (Anluohuaxian [18], Biejia-Ruangan [19], or FuZhengHuaYu [20]) on this group of patients. The primary outcome was cirrhotic decompensation (such as hepatic encephalopathy, ascites, or variceal bleeding), hepatocellular carcinoma, LT, and liver-related death. Biochemical response was assessed using Paris I [9], Paris II [5], Rotterdam [7], and Toronto [8] at 1 year after enrollment. The GLOBE score [11], which takes into account baseline measurements and parameters post-1 year of UDCA therapy, have been developed and implemented to forecast the long-term prognosis in patients with PBC and was calculated at enrollment. The GLOBE score cut-off values of 0.5 and 1.8 can distinguish PBC patients as low-, medium- and high-risk groups [15]. The study design was approved by the ethics committee of the Xijing Hospital of the Air Force Medical University.

### Treatment and follow-up

The baseline date referred to the date during the follow-up process at our center when LSM testing was conducted, at which point the patient must have been undergoing treatment with UDCA for a minimum of 6 months, either alone or in combination with fenofibrate. At this time, relevant clinical indicators for the included patient were at a relatively stable level. Follow-up visits were based on clinical and laboratory assessment every 6–12 months or sooner (depending on clinical status).

Elastography test: The LSM of a candidate with PBC was measured by trained physicians using FibroTouch [21]. The ultrasound probe was used to locate the site with uniform density in the liver parenchyma, avoiding cysts, bile ducts, and large blood vessels. The median LSM, in kPa, was taken as the LSM after ten or more consecutive successful measurements at the selected optimal site.

### Statistical analysis

We used SPSS version 26.0 (IBM) and R statistical software 4.13 (Tsinghua) for statistical analysis. We used the Shapiro–Wilk test to determine whether the data were normally distributed. Normally distributed continuous variables were expressed as mean ± standard deviation (SD); non-normally distributed variables were expressed as median (interquartile range, IQR). Comparison of the means of two continuous normally distributed variables was performed using the independent samples Student's t-test, and comparison of the medians of two continuous non-normally distributed variables was performed using the Mann–Whitney U test. Cox regression analyses were used to examine the relationship between prognostic indicators and the primary outcome. All variables with statistical significance in the univariate analysis were adjusted for in a multivariate model. The DeLong test was employed to compare the area under the receiver operating characteristic curve (AUC). The X-tile software was used to determine the optimal threshold for LSM. A statistically significant result was identified as a two-sided *p* < 0.05.

## Results

### Characteristics of patients receiving UDCA monotherapy at enrollment

**Fig. 1:**
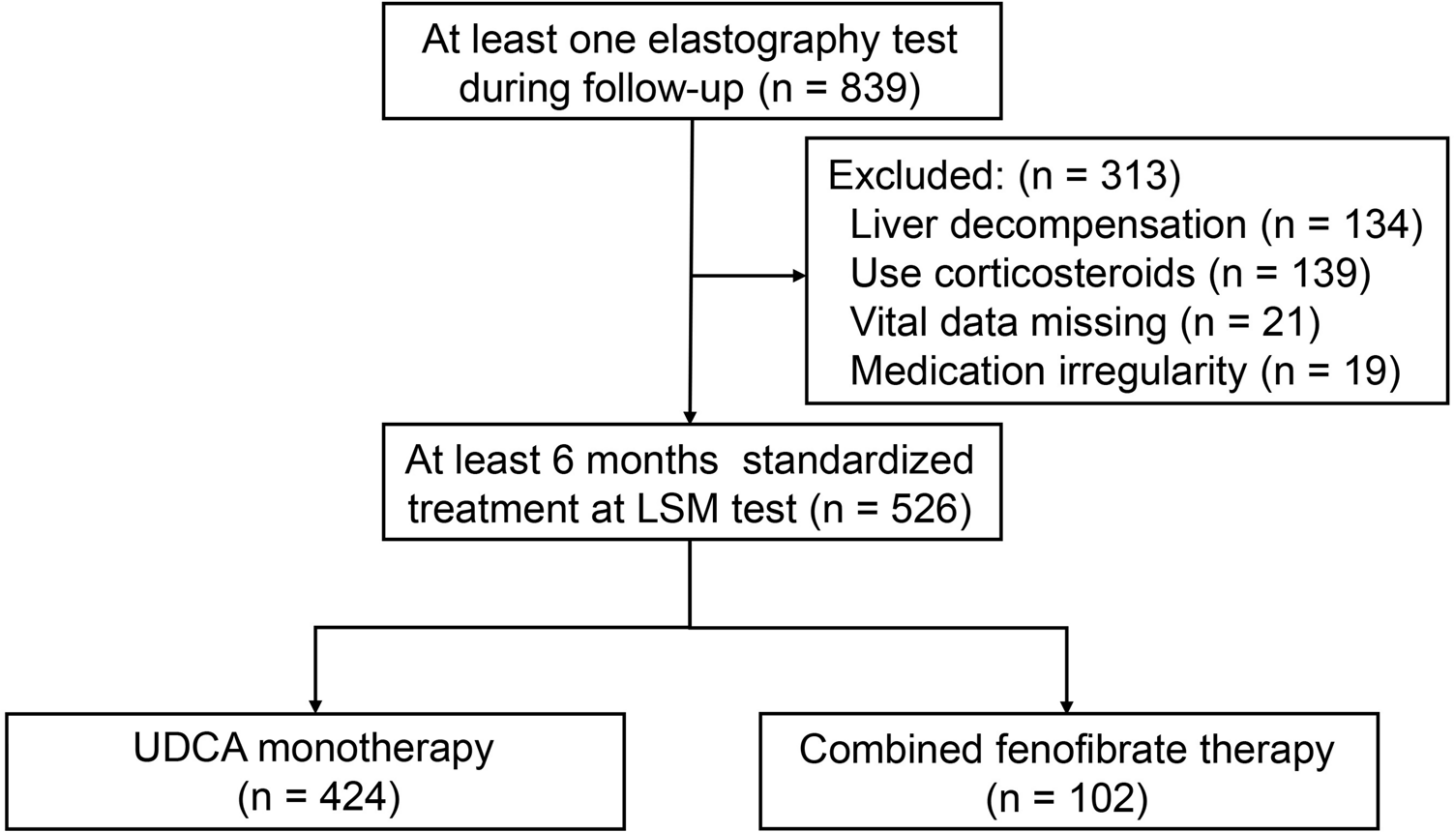
Case screening process and study design

Figure 1 presents the study flowchart. During the study period, 839 patients with at least one elastography test were screened for eligibility. A total of 526 patients who had received at least 6 months of standardized treatment at LSM testing were included in the study. Of the UDCA monotherapy group, the median follow-up time was 34 ± 20 months. 43 (10%) cases developed primary outcome, of which 32 (74%) had ascites, 1 (2%) had hepatic encephalopathy, 5 (12%) had variceal bleeding, 2 (5) had hepatocellular carcinoma, 1 (2) had LT, and 2 (5%) had liver-related death. Patients received UDCA for a median of 41 ± 39 months before enrollment.

The patients’ baseline characteristics are presented in Table 1. Their mean age was 54 ± 9 years, with 90% (381/424) being female and 85% (360/424) being anti-mitochondrial antibody (AMA)-positive. Thirty% (126/424) had fatigue and 17% (74/424) had pruritus. 34% (145/424) were anti-gp210 positivity and 21% (87/424) were anti-centromere antibody positivity. The median GLOBE score and LSM were 0.15 (− 0.38 to 0.75) and 9.25 (6.85–12.3), respectively. There were significant differences in the proportion of female gender (*p* = 0.027), follow-up time (*p* < 0.001), age (*p* = 0.013), platelet (PLT, *p* < 0.001), aspartate aminotransferase (AST, *p* < 0.001), total bilirubin (TBIL, *p* < 0.001), albumin (ALB, *p* < 0.001), LSM (*p* < 0.001), and GLOBE scores (*p* < 0.001) between groups with and without the primary outcome.

**Table 1:** Baseline indicators between patients receiving UDCA monotherapy with or without PO

Characteristics	Total (*n* = 424)	Without PO (*n* = 381, 90%)	With PO (*n* = 43, 10%)	*p* value
Age (years)	54 ± 10	53 ± 10	57 ± 9	0.013
Female (*n*, %)	381 (90)	347 (91)	34 (79)	0.027
Follow-up time (months)	34 ± 20	35 ± 20	25 ± 15	< 0.001
Fatigue (*n*, %)	126 (30)	108 (28)	18 (42)	0.066
Pruritus (*n*, %)	74 (17)	64 (17)	10 (23)	0.290
PLT × LLN	1.75 (1.22–2.24)	1.82 (1.33–2.30)	0.81 (0.61–1.18)	< 0.001
ALT × ULN	0.64 (0.43–1.01)	0.63 (0.40–0.98)	0.70 (0.44–1.10)	0.220
AST × ULN	0.89 (0.69–1.23)	0.86 (0.69–1.17)	1.14 (0.93–1.51)	< 0.001
ALB × LLN	1.10 (1.04–1.15)	1.11 (1.05–1.16)	0.99 (0.91–1.07)	< 0.001
TBIL × ULN	0.66 (0.52–0.90)	0.63 (0.50–0.83)	1.12 (0.89–1.66)	< 0.001
ALP × ULN	0.94 (0.69–1.30)	0.93 (0.69–1.27)	0.99 (0.78–1.66)	0.059
GGT × ULN	1.47 (0.67–3.10)	1.44 (0.67–3.04)	2.13 (0.48–3.69)	0.554
IgM × ULN	0.92 (0.59–1.40)	0.91 (0.58–1.41)	1.00 (0.69–1.38)	0.488
AMA (*n*, %)	360 (85)	323 (85)	37 (86)	0.826
Anti-gp210 antibody (*n*, %)	145 (34)	127 (33)	18 (42)	0.264
ACA (*n*, %)	87 (21)	71 (19)	16 (18)	< 0.001
LSM (kPa)	9.25 (6.85–12.3)	8.77 (6.67–11.48)	14.59 (12.05–17.46)	< 0.001
GLOBE score	0.15 (− 0.38 to 0.75)	0.07 (− 0.48 to 0.58)	1.30 (0.72–1.83)	< 0.001

Continuous variables were expressed as mean ± SD or median (interquartile range), while categorical variables were presented as *n* (%)

IgM available in 387 (91%) patients

PO, the primary outcome; UDCA, ursodeoxycholic acid; PLT, platelet count; ALT, alanine aminotransferase; AST, aspartate aminotransferase; ALB, albumin; TBIL, total bilirubin; ALP, alkaline phosphatase; GGT, gamma-glutamyl transpeptidase; IgM, immunoglobulin M; LSM, liver stiffness measurement; ACA, anti-centromere antibody; ULN, upper limit of normal; LLN, lower limit of normal

### Prognostic analysis to explore the superimposition effects of LSM on GLOBE

**Fig. 2:**
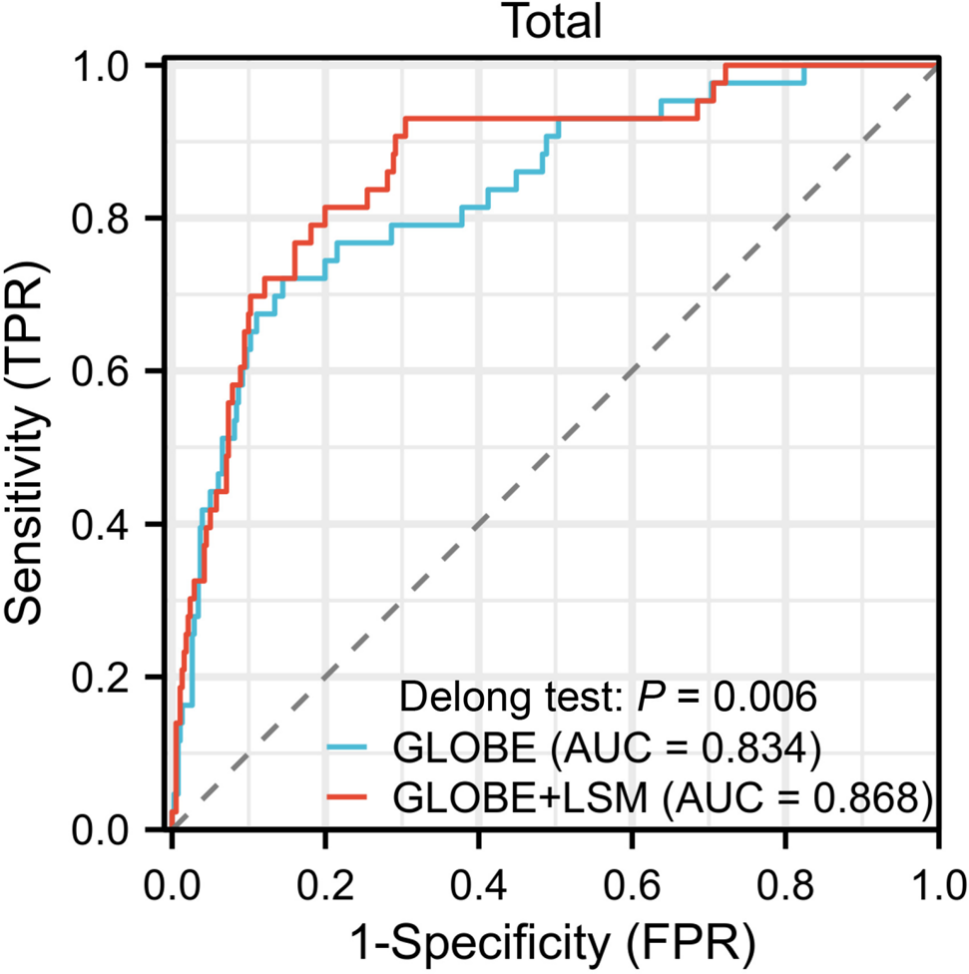
Receiver operating characteristic curves depicting the predictive efficacy of the fitted model (*y* = 1.325 × GLOBE score + 0.115 × LSM) and the GLOBE score for the primary outcome. LSM, liver stiffness measurement

All variables with statistical significance in the univariate analysis were adjusted for in a multivariate model. Multivariate Cox analysis demonstrated that GLOBE (hazard ratio [HR]: 3.762, 95% confidence interval [CI] 2.573–5.499, *p* < 0.001) and LSM (HR: 1.121, 95% CI 1.055–1.192, *p* < 0.001) were associated with LT-free survival. The fitted model was *y* = 1.325 × GLOBE score + 0.115 × LSM. The fitted model significantly improved the predictive efficacy of the primary outcome, with an area under the receiver operating characteristic curve (AUC) of 0.834 (0.767–0.900) for the GLOBE score and 0.868 (0.811–0.925) for the fitted model (*p* = 0.006, Fig. 2).

To further elucidate the additional prognostic effect of LSM on GLOBE, we risk-stratified the included population according to the GLOBE score. Previous study have found that the GLOBE scores of 0.5 and 1.8 can be used to classify populations into low, medium, and high-risk groups, and our cohort met this criterion (Supplementary Fig. 1) [15]. Comparison of AUC revealed additional prognostic effect of LSM on patients at low-risk as defined by GLOBE (0.777 [0.724–0.825] vs 0.642 [0.583–0.699], *p* = 0.001). LSM had no additional prognostic effect on medium and high-risk cases (0.811 [0.730–0.877] vs 0.745 [0.658–0.820], *p* = 0.157, and 0.576 [0.355–0.777] vs 0.527 [0.310–0.736], *p* = 0.839).

**Fig. 3:**
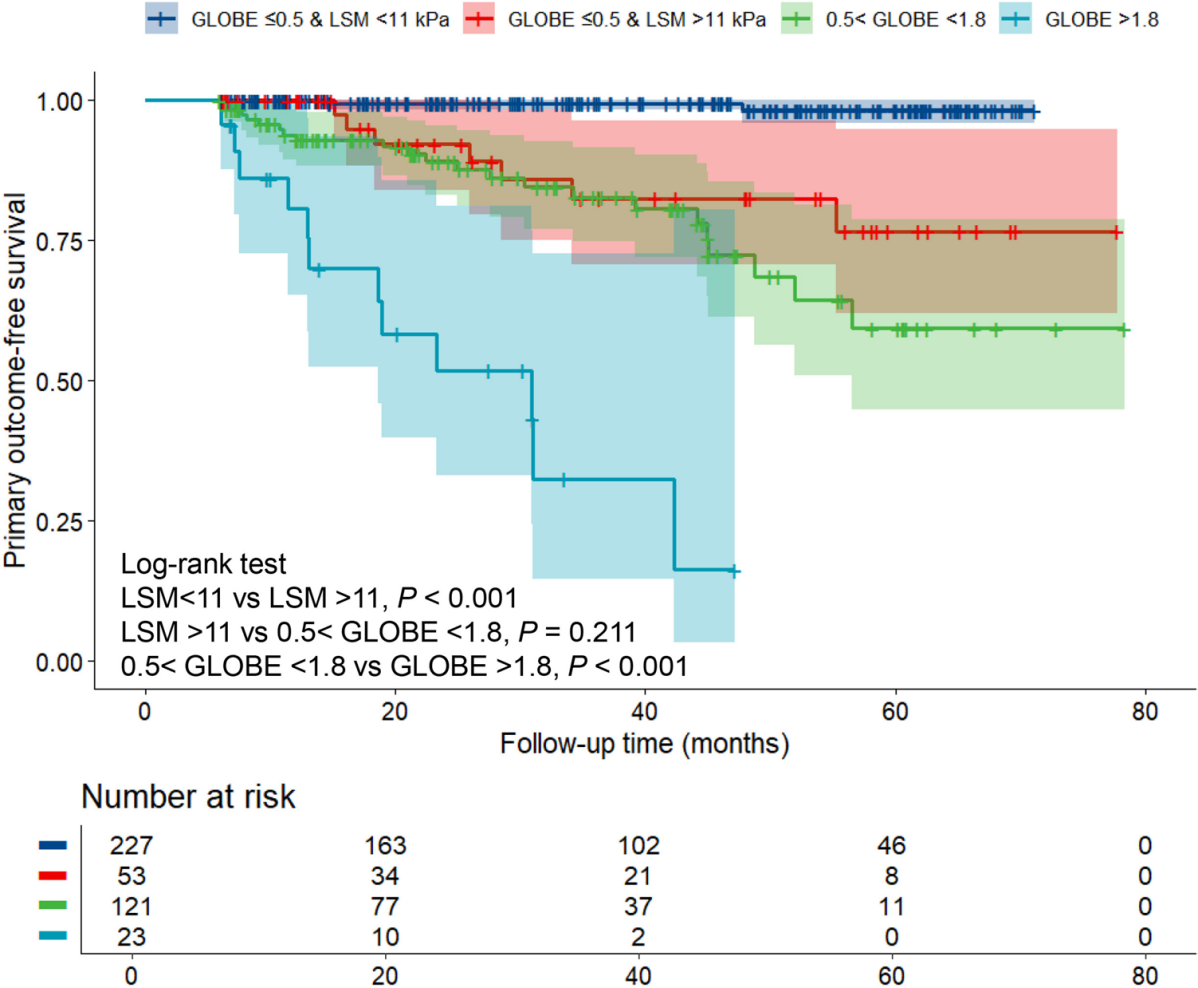
The Kaplan–Meier survival curves showing the prognosis of the included patients divided into four groups receiving ursodeoxycholic acid monotherapy

We performed prognostic analyses in the low-risk group defined by the GLOBE score, and univariate Cox regression found that LSM was associated with the primary outcome (HR: 1.292, 95% CI 1.132–1.475, *p* < 0.001), whereas GLOBE was not associated with the primary outcome (HR: 4.393, 95% CI 0.888–21.721, *p* = 0.070). The X-tile software was used to determine the optimal cut-off value of 11 kPa for LSM (Supplementary Fig. 2). When GLOBE < 0.5 and LSM > 11 kPa increased the incidence of the primary outcome by 15 times (7/53 [13%] vs 2/227 [1%], *p* < 0.001) compared to the GLOBE < 0.5 and LSM < 11-kPa group. Compared to biochemical response criteria (Paris 1, Paris 2, Toronto, or Rotterdam), LSM > 11 kPa predicted a higher positive predictive value, sensitivity, Youden index, and AUC for the primary outcome in low-risk patients with a GLOBE score < 0.5 (Supplementary Table 1). Based on the GLOBE score and LSM, we reclassified the included patients into four groups. The incidence of the primary outcome was 12/23 (52%) for high-risk and 22/121 (18%) for medium-risk. There was no difference between the medium and “low and LSM > 11 kPa” groups (Fig. 3).

### The LSM threshold of 11 kPa was suitable for patients receiving combined fenofibrate therapy.

Of the fenofibrate combined group, the median follow-up time was 40 ± 20 months. Twelve (12%) cases developed the primary outcome: 7 (58%) had ascites, 3 (25%) had variceal bleeding, and 2 (17%) had a liver-related death. Patients received fenofibrate for a median of 23 ± 21 months before enrollment.

The patients’ baseline characteristics are presented in Table 2. Their mean age was 54 ± 8 years, with 80% (83/102) being female and 84% (86/102) being AMA-positive. Forty eight% (49/102) had fatigue and 27% (28/102) had pruritus. 41% (42/102) were anti-gp210 positivity and 22% (22/102) were anti-centromere antibody positivity. The median GLOBE score and LSM were − 0.08(− 0.69 to 0.75) and 10.48(7.4–13.15), respectively. There were significant differences in PLT (*p* < 0.001), alanine aminotransferase (ALT, *p* = 0.003), AST (*p* < 0.001), alkaline phosphatase (ALP, *p* < 0.001), gamma-glutamyl transpeptidase (GGT, *p* = 0.005), TBIL (*p* < 0.001), ALB (*p* < 0.001), LSM (*p* < 0.001), and GLOBE scores (*p* < 0.001) between groups with and without the primary outcome.

**Table 2:** Baseline data between patients receiving fenofibrate combined therapy with or without PO

Characteristics	Total (*n* = 102)	Without PO (*n* = 90, 88%)	With PO (*n* = 12, 12%)	*p* value
Age (years)	54 ± 8	54 ± 8	52 ± 8	0.531
Female (*n*, %)	83 (80)	73 (81)	10 (83)	1.000
Follow-up time (months)	40 ± 20	40 ± 20	33 ± 21	0.257
Fatigue (*n*, %)	49 (48)	41 (46)	8 (67)	0.169
Pruritus (*n*, %)	28 (27)	24 (26)	4 (33)	0.290
PLT × LLN	1.79 (1.2–2.57)	2.01 (1.37–2.66)	1.19 (1.02–1.46)	< 0.001
ALT × ULN	0.78 (0.53–1.28)	0.74 (0.5–1.18)	1.23 (1.11–1.38)	0.003
AST × ULN	1.11 (0.86–1.82)	1.01 (0.8–1.43)	2.11 (1.86–2.49)	< 0.001
ALB × LLN	1.11 (1.08–1.17)	1.13 (1.09–1.17)	1.01 (0.98–1.08)	< 0.001
TBIL × ULN	0.66 (0.49–0.94)	0.62 (0.45–0.77)	1.33 (1.08–2.26)	< 0.001
ALP × ULN	0.84 (0.58–1.24)	0.76 (0.55–1.1)	1.97 (1.28–2.69)	< 0.001
GGT × ULN	2.14 (1.12–5.12)	1.93 (0.94–4.69)	4.94 (3.53–8.11)	0.005
IgM × ULN	0.75 (0.46–0.99)	0.73 (0.45–0.95)	0.85 (0.57–1.06)	0.309
AMA (*n*, %)	86 (84)	76 (84)	10 (83)	1.000
Anti-gp210 antibody (*n*, %)	42 (41)	33 (36)	9 (75)	0.026
ACA (*n*, %)	22 (22)	16 (18)	3 (25)	0.834
LSM (kPa)	10.48 (7.4–13.15)	10.01 (7.34–12.24)	15.6 (13.77–18.21)	< 0.001
GLOBE score	-0.08 (-0.69–0.75)	-0.17 (-0.73–0.37)	1.12 (0.91–1.76)	< 0.001

Continuous variables were expressed as mean ± SD or median (interquartile range), while categorical variables were presented as *n* (%)

IgM available in 101 (99%) patients

PO, the primary outcome; PLT, platelet count; ALT, alanine aminotransferase; AST, aspartate aminotransferase; ALB, albumin; TBIL, total bilirubin; ALP, alkaline phosphatase; GGT, gamma-glutamyl transpeptidase; IgM, immunoglobulin M; LSM, liver stiffness measurement; ACA, anti-centromere antibody; ULN, upper limit of normal; LLN, lower limit of normal

Univariate cox regression found that LSM was associated with the primary outcome (HR: 1.268, 95% CI 1.080–1.489, *p* = 0.004), whereas GLOBE was not associated with the primary outcome (HR: 333.074, 95% CI 0.305–364170.336, *p* = 0.104) in the low-risk group defined by GLOBE score. GLOBE < 0.5 and LSM > 11 kPa had higher incidence of the primary outcome (3/21 [14%] vs 0/47 [0%], *p* = 0.022) compared to the GLOBE < 0.5 and LSM > 11-kPa group. The incidence of the primary outcome was 3/3 (100%) for high-risk and 6/27 (22%) for medium-risk. There was no difference between the medium and “low and LSM > 11 kPa” groups (*p* = 0.353, Supplementary Fig. 3).

### The impact of antifibrotic drugs on the disease progression of patients with low-risk and LSM > 11 kPa

**Fig. 4:**
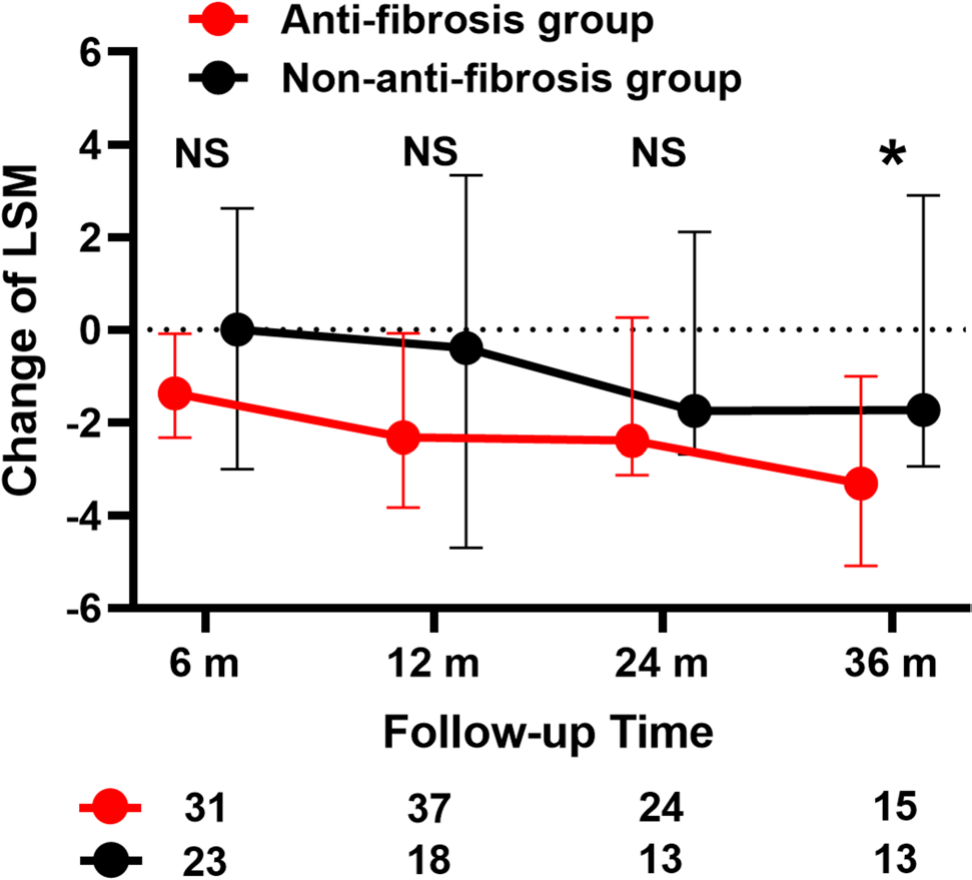
Changes in LSM at 6, 12, 24, and 36 months of follow-up between the anti-fibrosis and non-anti-fibrosis groups. Shown was median and interquartile range. The differences between these two groups at each time point were compared using Mann–Whitney U tests. LSM, liver stiffness measurement; m, months

A total of 75 patients with low-risk and LSM > 11 kPa were included, of whom 46 (61%) received antifibrotic therapy. Ninety one% (42/46) received FuZhengHuaYu treatment, 7% (3/46) received Anluohuaxian treatment, and 2% (1/46) received Biejia-Ruangan treatment. There were no differences between the anti-fibrosis and non-anti-fibrosis groups in terms of the included parameters (Supplementary Table 2). During follow-up, 11% (5/46) and 17% (5/29) achieved the primary outcome in the anti- fibrotic and non-anti-fibrotic groups, respectively (*p* = 0.659). To better elucidate the effects of antifibrotic drugs, we observed changes in LSM at different timepoints of follow-up. As shown in Fig. 4, the anti-fibrotic group had higher changes of LSM compared to the non-anti-fibrosis groups at 36 months of follow-up (− 3.31 [− 5.04 to − 1.52] vs − 1.74 [− 2.83 to 1.50], *p* = 0.046). No differences were found at 6 (− 1.37 [− 2.26 to − 0.12] vs 0 [− 2.75 to 2.51], *p* = 0.175), 12 (− 2.32 [− 3.59 to − 0.16] vs − 0.40 [− 3.77 to 2.67], *p* = 0.240), and 24 (− 2.40 [− 2.97 to − 0.17] vs − 1.75 [− 2.47 to 1.97], *p* = 0.189) months.

## Discussion

The research indicated that the combination of LSM and the GLOBE score improved prognostic prediction for patients. In particular, for patients in the low-risk group defined by GLOBE < 0.5, the prognosis was worse when LSM > 11 kPa, and their prognosis was consistent with that of patients in the medium-risk group defined by 0.5 < GLOBE < 1.8. This cut-off value also applied equally to patients treated in combination with fenofibrate, and antifibrotic therapy may delay the progression of liver stiffness in patients with GLOBE < 0.5 and LSM > 11 kPa.

Liver biochemical testing is the principal surrogate endpoint for death or the requirement for LT in most clinical trials [22]. The prognostic usefulness of biochemical endpoints in the specific context of combination therapy has not yet been fully demonstrated, and their predictive ability is only partially proven [15]. Since the evaluation of fibrosis staging has been demonstrated to have prognostic significance beyond the biochemical response to treatment, it has become necessary to include fibrosis staging in the PBC risk-stratification approach [23]. Consistent with Corpechot et al. [15], we showed in this investigation that LSM could enhance prognostic prediction when combined with the GLOBE score.

In clinical practice, the prognosis and treatment options for some patients treated with UDCA or combined fenofibrate who have well-controlled biochemical markers but a high degree of hepatic stiffness may be areas for further exploration. Based on the above considerations, we used the GLOBE score to categorize the included population into low, medium, and high-risk groups. Further studies found that LSM actually had a deleterious effect on the prognosis of patients in the low-risk group. When LSM was greater than 11 kPa, the incidence of the primary outcome was significantly higher in low-risk patients, and the incidence was essentially the same as that of patients in the medium-risk group. Meanwhile, LSM provided higher prognostic diagnostic efficacy in low-risk patients compared with biochemical response criteria. This prompted clinicians to pay adequate attention to low-risk patients as well, in particular when liver stiffness was high.

Fenofibrate was preferred and widely used in China for patients who were poor responders to UDCA, and studies by us and others have confirmed the beneficial efficacy of fenofibrate in these patients [24–26]. Criteria for the assessment of biochemical response in patients treated with combined fenofibrate are still lacking, and previous studies [24, 27] have used the risk scores for the prognostic assessment of these patients. In our included population, the risk of the primary outcome tended to increase in the GLOBE-defined low, medium, and high-risk groups. More importantly, consistent with UDCA monotherapy, we found that LSM > 11 kPa significantly increased the prognostic risk of fenofibrate-treated low-risk patients, with the incidence increasing to a rate comparable to that of the medium-risk group. This cut-off value may equally apply to patients treated with combined fenofibrate.

Strategies for further treatment of patients at low-risk but with LSM > 11 kPa may involve improving the degree of fibrosis. The role of traditional Chinese medicines [18–20] in antifibrosis has been confirmed in several studies, and the therapeutic mechanisms encompass the inhibition of hepatic stellate cell activation, attenuation of inflammation, protection of hepatocytes, suppression of angiogenesis in hepatic sinuses, facilitation of extracellular matrix degradation, and promotion of liver regeneration [28, 29], but whether they are effective in these particular patients remain to be elucidated. Due to the small sample size and short follow-up period, we did not find a difference in the incidence of the primary outcome between anti-fibrotic and non-anti-fibrotic groups during follow-up. However, LSM was lower in the antifibrotic group than in the non-antifibrotic group, suggesting that antifibrotic treatment may slow the progression of liver stiffness in these patients.

It is worth noting that our study has some limitations, such as the single-center retrospective design that may limit the generalizability of the findings, and introduces a potential selection bias that needs to be validated in future multi-center studies. In addition, we were unable to assess the additional prognostic role of LSM on the GLOBE score at the same time point after UDCA or combined fenofibrate therapy due to the limited included patients. Instead, we restricted inclusion of patients to those who had been treated with UDCA or combined fenofibrate for at least 6 months at baseline, at which moment the patients were in a relatively stable state of disease treatment. Given the small number of patients with paired liver biopsies and varying time intervals, it is difficult to evaluate the relationship between anti-fibrosis therapy and pathological results.

In conclusion, we found that GLOBE-defined low-risk patients receiving UDCA monotherapy or combined fenofibrate therapy with LSM > 11 kPa had a poor prognosis and that antifibrotic therapy may delay the progression of liver stiffness.

## Supplementary Information

Below is the link to the electronic supplementary material.

Supplementary file1 (DOCX 2280 KB)

## Data Availability

The data that support the findings of this study are available on request from the corresponding author.
