# Supplementary material for: Low-risk individuals with primary biliary cholangitis and significant liver stiffness: prognosis and treatment
Source: Hepatol Int. 2024 Dec 11;19(3):673–81. doi: 10.1007/s12072-024-10743-w (PMC12137486; doi:10.1007/s12072-024-10743-w)
Supplement: Supplementary file 1 — Supplementary file1 (DOCX 2280 KB) [file 12072_2024_10743_MOESM1_ESM.docx]

| **Supplementary Table 1** Comparison of the diagnostic efficacy of the fitted model and biochemical response criteria for the primary outcome in low-risk (GLOBE <0.5) patients | | | | | | | | |
| --- | --- | --- | --- | --- | --- | --- | --- | --- |
| Characteristics | Accuracy | PPV | NPV | sensitivity | Specificity | Youden | AUC | 95% CI |
| LSM> 11 kPa | 0.839 | 0.140 | 0.991 | 0.778 | 0.841 | 0.619 | 0.798 | 0.746-0.844 |
| Paris I | 0.900 | 0.048 | 0.969 | 0.111 | 0.926 | 0.037 | 0.519 | 0.458-0.578 |
| Paris Ⅱ | 0.814 | 0.061 | 0.974 | 0.333 | 0.830 | 0.164 | 0.582 | 0.522-0.640 |
| Toronto | 0.893 | 0.043 | 0.969 | 0.111 | 0.919 | 0.030 | 0.672 | 0.614-0.727 |
| Rotterdam | 0.886 | 0.129 | 0.980 | 0.444 | 0.900 | 0.345 | 0.515 | 0.455-0.575 |
| LSM, liver stiffness measurement; PPV, positive predictive value; NPV, negative predictive value; AUC, the area under the receiver operating characteristic curve; CI, confidence interval. | | | | | | | | |

|  | **Supplementary Table 2** Baseline data between patients with low-risk and LSM >11 kPa in the anti-fibrosis and non-anti-fibrosis groups. | | | | |
| --- | --- | --- | --- | --- | --- |
| Characteristics | | Total  (n = 75) | Anti-fibrosis  (n = 46, 61%) | Non-anti-fibrosis  (n = 29, 39%) | *P* value |
| Age (years) | | 51±9 | 51±9 | 51±8 | 0.997 |
| Female (n, %) | | 67(92) | 42(93) | 25(93) | 1.000 |
| Follow-up time (months) | | 35±21 | 32±20 | 40±22 | 0.139 |
| PLT×LLN | | 1.69(1.15-2.07) | 1.69(1.20-1.91) | 1.66(1.15-2.13) | 0.761 |
| ALT×ULN | | 0.73(0.46-1.05) | 0.73(0.53-1.05) | 0.73(0.43-1.02) | 0.543 |
| AST×ULN | | 0.97(0.74-1.26) | 0.97(0.80-1.23) | 0.96(0.72-1.31) | 0.825 |
| ALB×LLN | | 1.12(1.05-1.18) | 1.11(1.05-1.18) | 1.14(1.10-1.18) | 0.235 |
| TBIL×ULN | | 0.59(0.50-0.74) | 0.58(0.49-0.65) | 0.60(0.53-0.80) | 0.122 |
| ALP×ULN | | 0.94(0.60-1.16) | 0.88(0.65-1.15) | 1.00(0.56-1.22) | 0.694 |
| GGT×ULN | | 1.58(0.79-3.49) | 1.16(0.71-3.29) | 2.12(0.83-3.65) | 0.247 |
| IgM×ULN | | 0.89(0.57-1.30) | 0.94(0.66-1.31) | 0.72(0.51-1.05) | 0.153 |
| LSM (kPa) | | 60(83) | 38(84) | 22(81) | 1.000 |
| GLOBE score | | 12.85(11.65-14.55) | 12.72(11.64-14.34) | 13.15(11.94-17.04) | 0.567 |
|  | Continuous variables were expressed as mean ± SD or median (interquartile range), while categorical variables were presented as n (%).  PLT, platelet count; ALT, alanine aminotransferase; AST, aspartate aminotransferase; ALB, albumin; TBIL, total bilirubin; ALP, alkaline phosphatase; GGT, gamma-glutamyl transpeptidase; IgM, immunoglobulin M; LSM, liver stiffness measurement; ULN, upper limit of normal; LLN, lower limit of normal. | | | | |

**
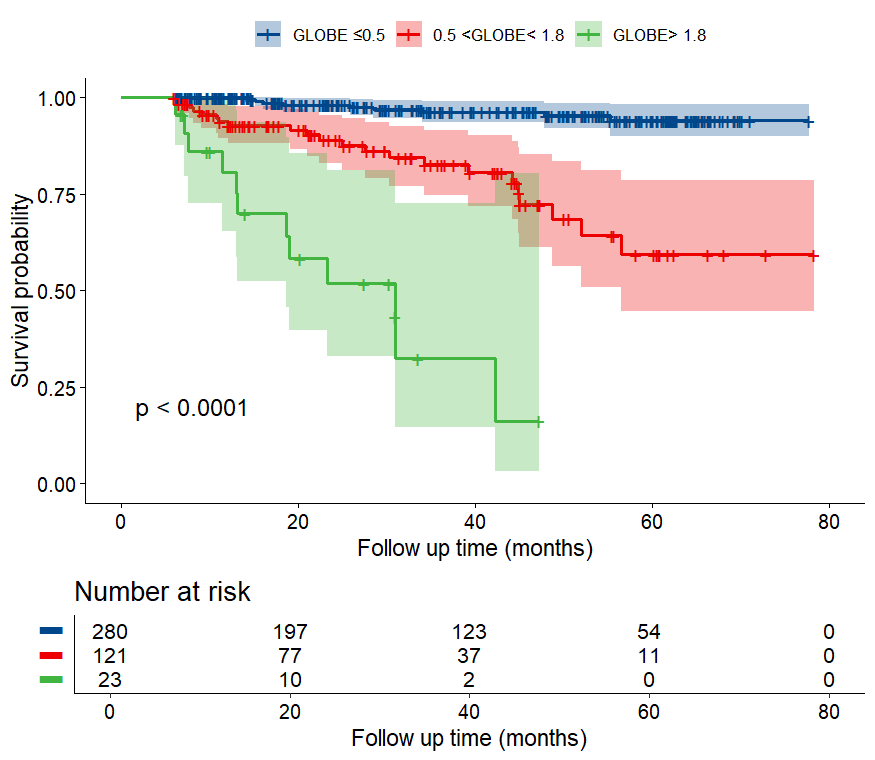
 Supplementary Figure 1** Kaplan-Meier survival curves indicating the GLOBE scores of 0.5 and 1.8 could classify enrolled patients receiving ursodeoxycholic acid monotherapy into low, medium, and high-risk groups.


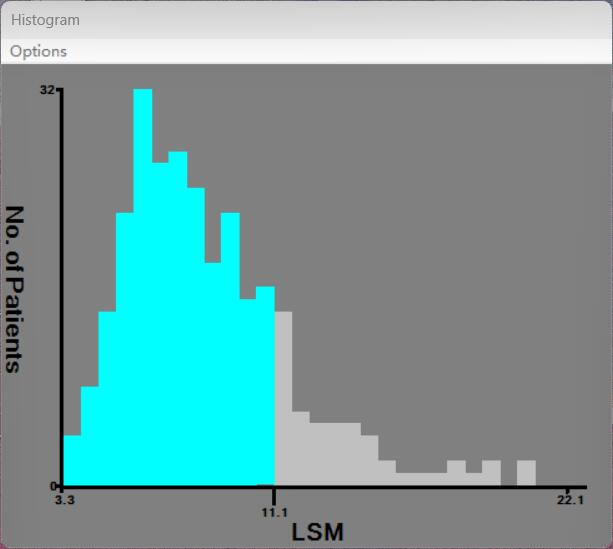

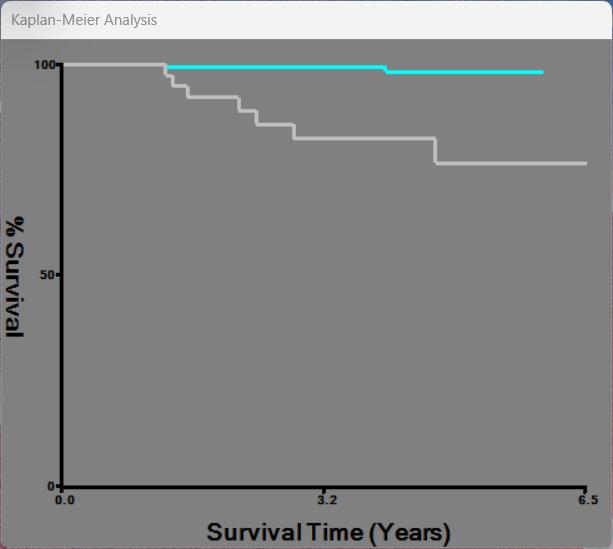


**
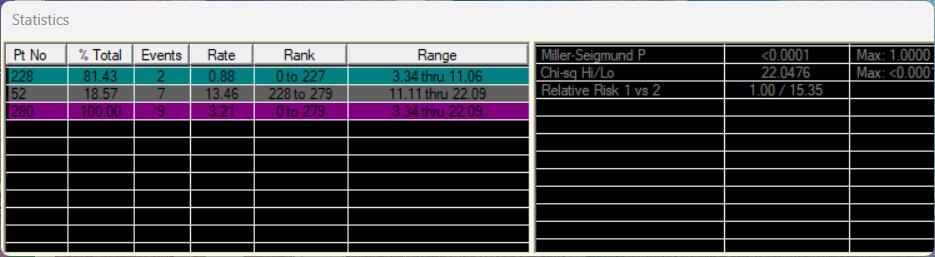
**

**Supplementary Figure 2** The X-tile software was used to determine the optimal cut-off value for LSM in the risk stratification of low-risk patients defined by the GLOBE score <0.5.


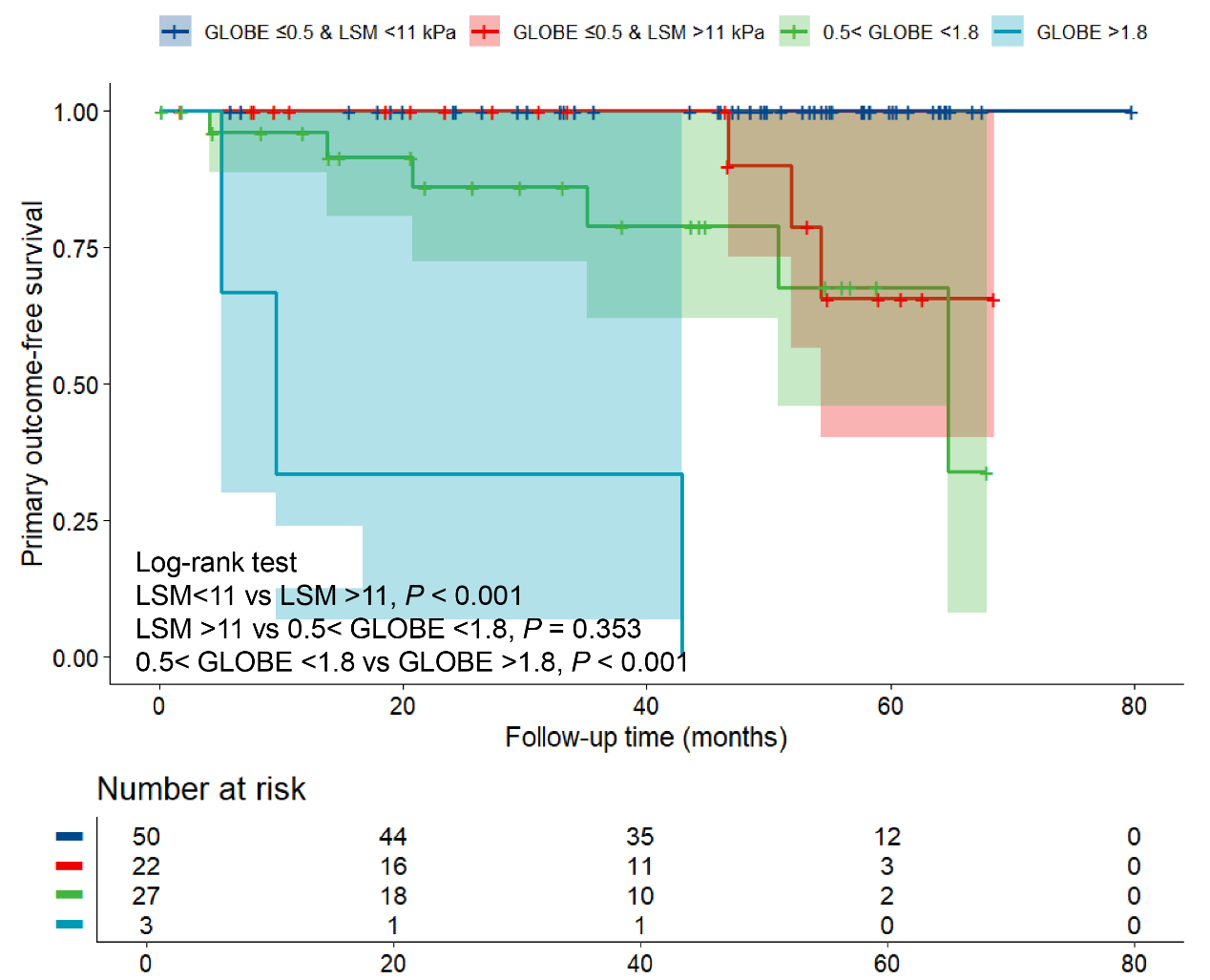
.

**Supplementary Figure 3** The Kaplan-Meier survival curves showing the prognosis of the included patients divided into four groups receiving combined fenofibrate therapy
